# Cerebrovascular events in patients with isolated anti-phosphatidyl-serine/prothrombin antibodies

**DOI:** 10.1007/s12026-021-09208-1

**Published:** 2021-07-10

**Authors:** Massimo Radin, Alice Barinotti, Silvia Grazietta Foddai, Irene Cecchi, Elena Rubini, Dario Roccatello, Elisa Menegatti, Savino Sciascia

**Affiliations:** 1 Center of Research of Immunopathology and Rare Diseases- Coordinating Center of Piedmont and Aosta Valley Network for Rare Diseases, Department of Clinical and Biological Sciences, S. Giovanni Bosco Hospital, University of Turin, Piazza del Donatore di Sangue 3, 10154 Turin, Italy; 2 School of Specialization of Clinical Pathology, Department of Clinical and Biological Sciences, University of Turin, Turin, Italy; 3 Nephrology and Dialysis, Department of Clinical and Biological Sciences, S. Giovanni Bosco Hospital and University of Turin, Turin, Italy

**Keywords:** Antiphospholipid syndrome, Anti-phosphatidyl-serine/prothrombin antibodies, Cerebrovascular events, Stroke, Thrombosis, Antiphospholipid antibodies

## Abstract

The interest of extra-criteria antiphospholipid antibodies is growing, especially in patients negative for conventional antibodies. In this study we aimed to assess the clinical utility of anti-phosphatidyl-serine/prothrombin antibodies (aPS/PT) testing in patients negative for Beta2-Glycoprotein 1(β2GPI)-dependent tests, for identifying antiphospholipid syndrome (APS) patients that developed cerebrovascular events (CVE). When screening APS patients attending our center, out of 119 aPS/PT IgG/IgM-positive patients, thus patients negative for aβ2GPI and aCL, 42 patients (35%) tested negative for β2GPI-dependent tests and were tested with thrombin generation assay (TGA). Ten patients (24%), with isolated aPS/PT IgG/IgM, had a history of CVE. Lupus anticoagulant (LA)-positive test was more frequently observed in patients with CVE (8/22 vs. 2/20; *p* = 0.045). Out of the 10 patients who experienced CVE, 3 patients were aPS/PT IgG positive (all LA positive), and 8 patients were aPS/PT IgM positive (6/8 LA positive). One patient was positive for both aPS/PT IgG and IgM. LA-positive patients had only high titers of aPS/PT IgG/IgM, all of them being ≥ 80 U/ml, while the 2 LA-negative patients were aPS/PT IgM positive with medium titers [40–60 U/ml]. LA-positive patients had significantly altered TGA profile when compared to those who were LA negative, considering all TGA parameters. LA-positive patients had significantly higher tLag (8.4 ± 3.3 min vs. 6.6 ± 1.8 min; *p* = 0.046), higher tPeak (14 ± 4.3 min vs. 11 ± 2.7 min; *p* = 0.015) and lower Peak (207 ± 152 nM vs. 356.3 ± 104.7 nM; *p* < 0.001) and lower AUC (2109.7 ± 1006.9 nM vs. 2772.5 ± 776.8 nM; *p* = 0.033). The use of aPS/PT might be of help in identifying patients with CVE and APS, as also confirmed by TGA testing.

## Introduction

Arterial thrombosis is one of the most life-threatening manifestation of the antiphospholipid syndrome (APS), an autoimmune disease characterized by the persistent presence of antiphospholipid antibodies (aPL) and development of clinical manifestations such as arterial and/or venous thrombosis and/or pregnancy morbidity [1].

A recent systematic review was performed with the support of Antiphospholipid Syndrome Alliance for Clinical Trials and International Networking (APS ACTION), investigating the overall prevalence of aPL in specific patients’ populations [2]. Data was retrieved by the analysis of 120 full-text papers, and the overall aPL frequency in patients with cerebrovascular events (CVEs) was estimated to be as high as 13.5% [2]. In order to investigate the prevalence of aPL in different subset of the general population, another very recent systematic review was conducted and focused on patients with CVEs and aged < 50 years old [3]. When focusing on 5217 patients and controls from 43 studies analyzing the frequency of aPL in young patients with CVEs, the overall aPL frequency was estimated to be as high as 17.4% (range 5–56%) for any CVE [3]. In particular, the presence of aPL increased the risk for developing CVEs by 5.48-fold (95% CI 4.42 to 6.79).

Laboratory criteria for APS include the assay tests for the presence of lupus anticoagulant (LA), anti-cardiolipin (aCL), and anti-β2-glycoprotein I (aβ2GPI) antibodies [1]. However, new autoantibodies specificities, which might be valuable for increasing the diagnostic accuracy and risk assessment strategies, are emerging. For instance, the use of anti-phosphatidylserine/prothrombin (aPS/PT) antibodies has been proposed, especially when criteria aPL are negative or inconclusive and for risk stratification assessment [4–6]. Indeed, further studies are needed to assess their role in the diagnostic algorithm for APS and for other clinical manifestations of the disease, such as CVEs.

Moreover, the study of thrombin generation could represent an additional novel technique that could have the potential to improve the thrombotic risk assessment of this particular category of patients [7–9]. A significant number of studies highlighted that altered thrombin generation may lead to pathologic processes, mainly hemorrhagic or thrombotic diseases. The evaluation of an individual’s thrombin-generation potential could serve as a useful estimator of the total coagulation potential.

The integration of laboratory testing and clinical information can potentially ameliorate the thrombotic risk assessment of patients, as demonstrated by scoring systems such as the Global Antiphospholipid score (GAPSS) [10, 11].

In this study, we aimed to evaluate the clinical utility of aPS/PT testing and thrombin generation assay (TGA), in patients negative for β2GPI-dependent tests (aβ2GPI and aCL antibodies), in patients who experienced CVEs.

## Methods

### Patients

All aPS/PT IgG/IgM persistently positive patients (defined as aPS/PT IgG/IgM ≥ 40 U/ml on at least 2 occasions ≥ 12 weeks apart) that presented at San Giovanni Bosco Hospital in the last 2 years were chart-reviewed.

Indication for aPS/PT testing were (a) high clinical suspicion of APS and (b) immunological assessment of patients with systemic lupus erythematosus (SLE).

Out of 119 aPS/PT IgG/IgM-positive patients, 42 patients (35%) were enrolled for the sake of this study because negative for β2GPI-dependent tests, thus patients negative for aβ2GPI and aCL.

Clinical and laboratory characteristics were retrospectively collected.

### Autoantibodies testing

The IgG/IgM isotype for aCL, aß2GPI, and aPS/PT antibodies were detected by commercial ELISA (Inova Diagnostics, Inc., San Diego, CA, USA). LA was tested with the detection of two different reagents, used as screening and confirmatory tests, Silica Clotting Time HemosIL and dRVVT Screen and Confirm HemosIL, respectively (Instrumentation Laboratory, Bedford, MA, USA), following the ISTH guidelines [12].

### Thrombin generation assay (TGA)

TGA is able to quantify the total amount of thrombin generated in a plasma sample, serving as an estimator of the total coagulation potential. TGA was performed by a commercially available assay kit (Technothrombin TGA kit, Techonoclone, Vienna, Austria) on a fully automated, computer-controlled micro-plate-reader and a specially adapted software (Technothrombin TGA, Vienna, Austria)[9]. Briefly, the concentration of thrombin generated in the plasma sample has been registered over time resulting in a thrombin generation curve, allowing the estimation of several parameters, including: the time interval between the addition of the triggers and the beginning of the reaction (Lag time — tLag), the highest amount of thrombin generated (Peak), the time to reach this Peak (time to Peak — tPeak) and the total amount of thrombin generated (Area under the curve — AUC).

### Statistical analysis

Categorical variables are presented as number (%) and continuous variables are presented as mean (S.D.). The significance of baseline differences was determined by the Chi-squared test, Fisher’s exact test or the unpaired t-test, as appropriate. A two-sided *P* value < 0.05 was statistically significant. All statistical analyses were performed using SPSS version 26.0 (IBM, Armonk, NY, USA).

## Results

A total of 42 patients were enrolled in the study. Demographic and laboratory characteristics of the patients are summarized in Table 1.

**Table 1:** Demographic and laboratory characteristics of the patients enrolled in the study

	All (n = 42)		LA positive (n = 22)	LA negative (n = 20)
Anagraphic				
Mean age (± S.D.) at data collection		42.2 ± 12.8	43.1 ± 10.4	46.5 ± 10.5
Sex (females), n (%)		38 (90.5%)	19 (86.4%)	19 (95%)
Secondary autoimmune diagnosis, n (%)		SLE 7 (16.7%)	SLE 4 (18.2%)	SLE 3 (15%)
APS, n (%)		20 (47.6%)	13 (59.1%)	7 (35%)
aPL asymptomatic, n (%)		22 (52.4%)	9 (40.9%)	13 (65%)
Clinical manifestations of APS patients (n, 20)				
Thrombosis, n (%)	17 (40.5%)		12 (54.5%)	5 (25%)
Arterial thrombosis, n (%)	11 (26.2%)		10 (45.5%)	1 (5%)
Venous thrombosis, n (%)	8 (19%)		4 (18.2%)	4 (20%)
Cerebrovascular events, n (%)	10 (23.8%)		8 (36.4%)	2 (10%)
Deep vein thrombosis, n (%)	4 (9.5%)		2 (9.1%)	2 (10%)
Pulmonary embolism, n (%)	2 (4.8%)		1 (4.5%)	1 (5%)
Myocardial infarction, n (%)	3 (7.1%)		2 (9.1%)	1 (5%)
Pregnancy morbidity, n (%)	6 (14.3%)		3 (13.6%)	3 (15%)
aPS/PT testing				
aPS/PT IgG + , n (%)	13 (31%)		8 (36.4%)	5 (25%)
aPS/PT IgM + , n (%)	37 (88.1%)		20 (90.9%)	17 (85%)

*S.D.* standard deviation; *N/A* not applicable; *APS* antiphospholipid syndrome; *PAPS* primary APS; *SAPS* secondary APS; *aPS/PT* anti-phosphatidyl-serine/prothrombin antibodies; *Ig* immunoglobulin

Briefly, 10 patients out of 42 (24%) were positive for aPS/PT IgG/IgM and negative for aβ2GPI-dependent tests and had a history of CVEs. More in detail, 6 patients experienced an episode of ischemic stroke (one of them during a catastrophic APS), 3 patients had a cerebral venous sinus thrombosis, and 1 patient experienced a transient ischemic attack.

When looking at aPS/PT positivity, out of the 10 patients who experienced CVEs, 3 patients were IgG positive (all of them LA positive), and 8 patients were IgM positive (6 of them LA positive). One patient was positive for both aPS/PT IgG and IgM.

Interestingly, LA-positive patients who experienced CVEs had only high titers of aPS/PT IgG/IgM, all of them being ≥ 80 U/ml, while the 2 LA-negative CVEs patients were aPS/PT IgM positive with medium titers [40–60 U/ml].

When considering all 42 patients, those who were LA positive had significantly higher levels of aPS/PT IgM (155.9 ± 144.4 vs. 77.8 ± 52.7 U/ml, respectively; *p* = 0.021) and higher levels of aPS/PT IgG, but failed to reach a statistical significance (69.3 ± 85.9 vs. 33.8 ± 42.2 U/ml, respectively; *p* = 0.095).

**Fig. 1:**
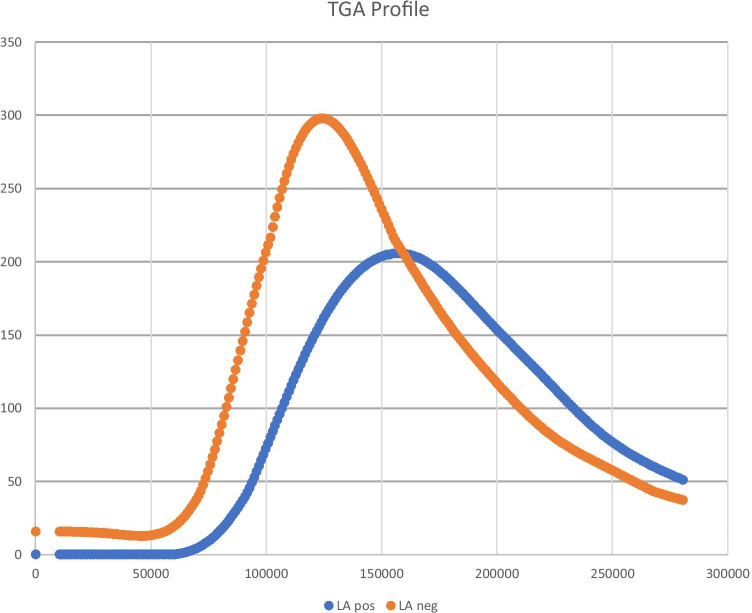
Representative thrombin generation assay profile of the patients enrolled in the study based on their lupus anticoagulant status. TGA – thrombin generation assay; APS – antiphospholipid syndrome; aPL – antiphospholipid antibodies; HC – healthy controls; LA – lupus anticoagulant

Interestingly, also when considering all 42 patients, those who were LA-positive experienced significantly more CVEs (8 patients out of 22 vs. 2 patients out of 20; *p* = 0.045) and had significantly altered TGA profile, when compared to those who were LA-negative, considering all TGA parameters. More in detail, LA-positive patients had significantly higher tLag (8.4 ± 3.3 min vs. 6.6 ± 1.8 min; *p* = 0.046), higher tPeak (14 ± 4.3 min vs. 11 ± 2.7 min; *p* = 0.015), lower Peak (207 ± 152 nM vs. 356.3 ± 104.7 nM; *p* < 0.001) and lower AUC (2109.7 ± 1006.9 nM vs. 2772.5 ± 776.8 nM; *p* = 0.033). TGA profiles of the two groups (based on their LA status) are illustrated in Fig. 1.

## Discussion

By the end of 2021, heart disease and stroke will become the leading cause of both death and disability worldwide, with the number of fatalities projected to increase to over 20 million a year and by 2030 to over 24 million a year [13]. Around 10% of all thrombotic CVE occur in young population and, in a large proportion of those, the trigger remains undetermined, classifying those CVE accidents as cryptogenic [14]. According to WHO estimates, 15 million people each year are affected by strokes, and 5 million are left permanently disabled [15]. Therefore, the prevention of new cryptogenic events, especially in young patients, must be a priority, and the identification of high-risk patients could optimize treatment and follow-up. While CVE-associated mortality has decreased in the last decades, stroke remains a major cause of death and disability in the general population [16], and it also represents one of the most life-threatening manifestations of APS.

When focusing on stroke management of patients without APS, besides the intervention on modifiable risk factors (e.g., hypertension, smoking habit, diet, diabetes, obesity, alcohol consumption, cardiac diseases, and physical inactivity), the mainstay is represented by the dual or mono-antiplatelet therapy, based on the use of drugs such as aspirin, clopidogrel, and dipyridamole. Only in rare cases, the anticoagulant therapy, the mainstay of APS management, is considered outside of APS, principally when patients are affected by atrial fibrillation [17–19]. APS in cryptogenic events is not as rare as one could imagine. In fact, in a study by Sciascia et al., it has been highlighted that aPL frequency in young patients (< 50 years old) who experienced CVE is estimated to be as high as 17.4% (5–56%) and that the aPL positivity increases the risk of CVE by 5.48-fold [3]. The correct identification of the cause of a CVE, especially if induced by an aPL-related event, is of critical importance, because the therapeutic approaches differ, and the prognosis radically changes.

The laboratory criteria for APS diagnosis comprehend the detection of at least one of the following aPL: LA, β2GPI, and aCL (at least two determinations, 12 weeks apart) [1]. Nonetheless, some “extra-criteria aPL” are emerging as potential additional specificities to be considered when diagnosing APS, in particular aPS/PT [5, 20, 21]. The growing importance of these aPL relies in the fact that some patients show a borderline condition: it is not uncommon to find individuals at high clinical suspicion of APS but negative for the three criteria aPL or, in some cases, negative for the solid assays (β2GPI and aCL) and showing an inconclusive LA test. In these cases, testing for “extra-criteria aPL” seem to be valuable approach that can be important in the clinical practice, for instance, to improve the diagnostic accuracy and when deciding on patients’ management.

In this study we focused on APS patients who experienced CVE and that were negative for the two solid assays for criteria-aPL detection and positive for aPS/PT IgG/IgM. When considering their LA status, LA-positive patients experienced significantly more CVEs and had only high titers of aPS/PT IgG/IgM (≥ 80 U/ml), while the LA-negative patients were aPS/PT IgM positive with medium titers (40–60 U/ml). Moreover, when analyzing also the TGA profile of all 42 patients, those who were LA positive had a significantly altered thrombogram when compared to those who were LA negative, considering all TGA parameters. Focusing on this latter aspect, even if the trend of LA-positive patients thrombogram seem to be characteristic of a hypocoagulable state, we have to consider LA paradoxical effect. LA is known to act as a pro-coagulant agent in vivo, while in vitro it causes the prolongation of the laboratory coagulation tests time because of its capacity to bind phospholipids. With this in mind and considering that also the TGA employs phospholipids to induce the beginning of the reaction, it does not seem surprising to observe the same trend when analyzing the TGA outcomes of this group of patients.

Overall, these results are in line with the well-accepted consideration according which among aPL, the positivity for LA represents one of the major risk factors for thrombotic events development, as well as the positivity for all the three criteria-aPL [22–25]. However, despite significant progresses in LA testing thanks to the updated guidelines of the ISTH [12], this assay still suffers from some shortcomings and it remains much more labor intensive and complicated to perform compared to immunoassays [26]. The relationship between LA and aPS/PT requires some further comments. Soon after the first description of prothrombin as part of the phospholipid binding protein family in 1959 [27], it was speculated that prothrombin could act as cofactor for LA. Subsequent studies [28] showed that anti-prothrombin antibodies can have LA activity. Similarly, it was also shown that the IgG fraction containing LA activity bound to the phospholipid–prothrombin complex. Those studies supported the hypothesis that both prothrombin and β2GPI can be target for autoantibodies with LA activity. More recently, Atsumi et al. [29] when investigating a cohort of 265 patients who visited an autoimmune disease clinic showed that IgG aPS/PT strongly correlated with the presence of LA as detected using the dilute Russell viper venom time test (OR 38.2, 95% CI 13.4–109.1). Those findings are in line with a subsequent study, showing that aPS/PT are frequently found in patients with LA, but their association with thrombosis seems to be independent of the presence of LA. Moreover, in a recent work Cifù et al. [30], treating monocytes and endothelial cells with the IgG fraction of aPS/PT isolated from APS patients, showed how aPS/PT display a pivotal role in the pathogenesis of the thrombotic events associated with APS.

Our study further confirms the abovementioned observations, showing that the association of LA and aPS/PT might confer an increased risk for CVEs, even when β2GPI-dependent tests are negative. Besides, the presence of LA in patients without aβ2GPI-antibodies could be explained by the presence of aPS/PT antibodies, as previously suggested [30, 31]. Importantly, aPS/PT testing in this study allowed the identification of patients suffering from APS, negative for aβ2GPI-dependent tests.

Some limitations should be acknowledged. First, the retrospective nature of the study could potentially limit the reproducibility of its results. Second, the sample size was limited; however one should bear in mind that APS is a rare disease, especially considering the low rate of patients with positive aPS/PT and negative aβ2GPI-dependent tests. Finally, patients’ samples have been tested for LA briefly before the ISTH recommendation update; thus LA outcomes are based on 2009 recommendations.

The results of our study might contribute to highlight the importance of aPS/PT in CVEs onset associated with APS and, more in general, in APS pro-thrombotic tendency. The clinical observations indeed were confirmed by TGA testing, showing a pro-thrombotic status in patients with aPS/PT, especially when LA was also positive. aPS/PT testing might be an added tool for risk stratification strategies, in particular when considering patients negative for the aβ2GPI-dependent tests or showing an inconclusive LA outcome, especially considering the abovementioned shortcomings characterizing this functional assay.

To date, correctly identifying the causes of cryptogenic CVEs in the general population is still an unmet need, and aPS/PT assay and TGA outcomes could help the treating clinicians in the near future.

## Data Availability

The datasets used and/or analyzed during the current study are available from the corresponding author on reasonable request.

## Abbreviations

aPS/PT: Anti-phosphatidyl-serine/prothrombin antibodies
APS: Antiphospholipid syndrome
CVE: Cerebrovascular event
TGA: Thrombin generation assay
β2GPI: Beta2-Glycoprotein 1
LA: Lupus anticoagulant
aCL: Anticardiolipin
aPL: Antiphospholipid antibodies
APS ACTION: Antiphospholipid Syndrome Alliance for Clinical Trials and International Networking
GAPSS: Global AntiPhoSpholipid Score
SLE: Systemic lupus erythematosus
tLag: Lag time
tPeak: Time to Peak
AUC: Area under the curve
